# Prognostic value and immunological roles of GPX3 in gastric cancer

**DOI:** 10.7150/ijms.85253

**Published:** 2023-09-04

**Authors:** Qijin He, Na Chen, Xu Wang, Ping Li, Limin Liu, Zheng Rong, Wentian Liu, Kui Jiang, Jingwen Zhao

**Affiliations:** Department of Gastroenterology and Hepatology, Tianjin Medical University General Hospital, Tianjin Institute of Digestive Diseases, Tianjin Key Laboratory of Digestive Diseases, No. 154 Anshan Road, Tianjin, 300052, China.

**Keywords:** gastric cancer, GPX3, ceRNA, prognosis, immunotherapy

## Abstract

**Objective:** The prognosis for gastric cancer (GC), a prevalent tumor of the digestive system, is unfavorable. The involvement of glutathione peroxidase 3 (GPX3) in tumorigenesis is significant, yet its specific role in GC remains insufficiently investigated. Thus, the aim of this study was to determine the potential impact of GPX3 on GC and elucidate the underlying mechanism.

**Methods:** The expression and survival of GPX3 in GC were analyzed using TCGA data. Additionally, the GPX3 mRNA and protein levels in GC were also assessed using datasets from GTEx, GEPIA, and HPA. A total of 38 pairs of GC tissues, along with their adjacent normal tissues, were collected from the Tianjin Medical University General Hospital, accompanied by detailed clinical information. The expression levels of GPX3 were subsequently determined for the purpose of validation. Following expression, correlation, and survival analyses, we proceeded to investigate the upstream non-coding RNA (ncRNA) of GPX3 using starBase and miRNet. Additionally, the co-expression networks of GPX3 were examined based on LinkedOmics. Lastly, we explored the correlation between GPX3 and immune cell infiltration, as well as the biomarkers of immune cells and immune checkpoints in GC. Furthermore, the GDSC database offered valuable drug sensitivity information.

**Results:** A lower expression of GPX3 was observed in individuals with GC, while a higher expression of GPX3 was associated with a poorer prognosis. The DUBR/hsa-miR-502-3p/GPX3 pathway was identified as the most promising upstream ncRNA pathway related to GPX3 in GC. GO and KEGG enrichment analysis revealed that GPX3 expression was linked to coagulation cascades and cell locomotion. Furthermore, GPX3 levels in GC were positively correlated with immune cell infiltration, immune cell biomarkers, and immune checkpoint expression. The group with low GPX3 expression also exhibited increased sensitivity to 5-fluorouracil, doxorubicin, and other drugs.

**Conclusions:** Collectively, we hypothesized that the potential involvement of non-coding RNAs in the downregulation of GPX3 could contribute to the inhibition of tumor formation during the malignant transition from gastritis to GC. Nevertheless, it was plausible that GPX3 may also facilitate tumor progression to advanced stages by promoting immune cell infiltration and activating immune checkpoints.

## Introduction

According to recent statistics, gastric cancer (GC) is ranked fifth and fourth in terms of morbidity and mortality among all malignancies 1. In the year 2020, the global incidence of GC exceeded 1 million new cases, leading to approximately 769,000 deaths and imposing a significant burden on global health 1. Radical surgery has emerged as the most efficacious approach for treating GC, with patients diagnosed at an early stage experiencing a notable 5-year survival rate of 95% due to advancements in surgical techniques, chemotherapy, and radiotherapy 2. However, despite this fact, a significant majority of patients, exceeding 70%, ultimately progress to advanced GC as a consequence of the insidious nature of early GC, leading to unfavorable prognosis and diminished survival rates. Consequently, there is an urgent need for therapeutic targets or prognostic biomarkers in the field of GC.

Glutathione peroxidase 3 (GPX3) is a key enzyme in the removal of hydrogen peroxide and other reactive oxygen species from cells during oxidative stress 3,4. The kidney serves as the site of GPX3 synthesis and actively releases it into extracellular fluids, such as plasma, eye chamber water, and amniotic fluid 5,6. Researchers have established a correlation between abnormalities in GPX3 expression and digestive system cancers 7. It has been verified that GPX3 is consistently underexpressed in various tumor tissues, primarily due to the methylation of the GPX3 gene, resulting in decreased GPX3 activity 8. In colorectal cancer, GPX3 methylation reduced GPX3 expression and increased oxaliplatin and cisplatin sensitivity 9. Furthermore, in patients over the age of 60 with GC, GPX3 hypermethylation was associated with a shorter recurrence time 10. The silencing of GPX3 gene expression in esophageal squamous cell carcinoma (ESCC) was attributed to CpG island methylation within the GPX3 gene promoter region, suggesting its potential as a biomarker for ESCC 11.

The analysis of the human genome has revealed that a significant portion of the genome consists of non-protein-coding DNA, with only approximately 3% of genes being responsible for protein synthesis 12,13. Non-coding RNA (ncRNA) encompasses a group of RNA molecules that lack the ability to encode proteins but play crucial roles in the pathophysiological regulation of various cellular processes such as proliferation, differentiation, apoptosis, infection, immune response, and are closely associated with the development and progression of malignant tumors and chronic diseases 14-16. Several subtypes of ncRNA exist, including long non-coding RNA (lncRNA), microRNA (miRNA) and circular RNA (circRNA). Research has demonstrated that lncRNAs possess a sponge adsorption effect on miRNAs and can competitively bind to them as competitive endogenous RNA (ceRNA), consequently impacting the binding of miRNAs to messenger RNA (mRNA) and regulating the initiation, progression, and metastasis of tumors 17. One study revealed that the upregulation of miR-1696 could impede the generation of reactive oxygen species (ROS) by suppressing the expression of GPX3, thereby hindering the formation of neutrophil extracellular traps 18. A separate investigation revealed that miR-483-5p was upregulated in cisplatin-induced acute kidney injury, leading to the promotion of oxidative stress and tubule cell apoptosis through negative regulation of GPX3 19. Furthermore, in non-small cell lung cancer, miR-196a facilitated tumor progression by activating the JNK pathway via the down-regulation of GPX3 20. Nevertheless, there remains a dearth of comprehensive research regarding the expression, prognostic implications, and potential ceRNA mechanism associated with GPX3 in GC.

In this study, we performed expression and survival analyses for GPX3 in GC. To the best of our knowledge, this is the first study on these lines. The findings revealed a low expression of GPX3 in GC, which was found to be associated with an unfavorable prognosis. Subsequently, we explored the involvement of miRNAs and lncRNAs in the regulation of GPX3 in GC. The DUBR/hsa-miR-502-3p/GPX3 axis emerged as the most probable upstream pathway. Furthermore, the expression of GPX3 exhibited a correlation with immune cell infiltration and immune checkpoint activity in GC. Our study contributed additional evidence to support the involvement of GPX3 in the progression of GC, indicating that reduced expression of GPX3 is linked to prognosis and immune invasion in GC patients.

## Materials and methods

### Data collection and expression analysis

The TCGA dataset, accessible at https://portal.gdc.cancer.gov, was employed to conduct a comparative analysis of GPX3 expression in GC. The current-release (V8) GTEx datasets, available at https://www.gtexportal.org/home/datasets, provided a collection of normal tissues for reference. Both the TCGA and GTEx datasets were utilized for the examination of lncRNA expression. The microarray data, specifically GSE27342 (Platform: GPL5175) and GSE33335 (Platform: GPL5175), were obtained from the GEO database at http://www.ncbi.nlm.nih.gov/geo.

### Prognosis analysis

The analysis conducted in this study utilized the TCGA dataset. In order to assess the correlation between the gene and survival in GC, four measures were employed: overall survival (OS), disease-specific survival (DSS), disease-free survival (DFS), and progression-free survival (PFS). Kaplan-Meier curves were generated and p-values, hazard ratios (HR), and 95% confidence intervals (95% CI) were calculated using log-rank tests and univariate cox proportional hazards regressions.

### GEPIA database analysis

GEPIA database (http://gepia.cancer-pku.cn/index.html) contains gene expression profiles relating to cancer and normal tissues. Our analysis of GPX3 expression and prognosis in the GC was conducted with GEPIA.

### Immunohistochemistry (IHC) staining

An investigation was conducted to compare the expression of GPX3 at the protein level, utilizing immunohistochemistry (IHC) images obtained from the Human Protein Atlas (HPA) database (www.proteinatlas.org/). The staining procedure involved the use of anti-body CAB069456.

### Clinical samples collection

A total of 38 pairs of tissue samples, comprising both GC tissues and adjacent normal tissues, were collected from patients diagnosed with primary GC at Tianjin Medical University General Hospital. The samples were stored at a temperature of -80°C until RNA extraction. Additionally, relevant clinical characteristics including patient age, gender, depth of tumor invasion, distant metastasis, and lymph node status were gathered and subjected to analysis. Prior to the commencement of the study, all patients provided informed consent on a voluntary basis. Moreover, this experiment was supported by the Ethics Committee of Tianjin Medical University General Hospital (Approval number: IRB2023-WZ-061).

### Quantitative real‐time PCR (qRT-PCR)

The whole RNAs were isolated from cells or tumor tissues using the Trizol method. A reverse transcription kit (Vazyme, China) was employed to reverse transcribe the total RNA. The RNA concentration of each sample was assessed individually, and 1μg of total RNA was added to each reverse transcription system. During the reverse transcription process, the 4 × gDNA wiper Mix effectively eliminated any remaining genomic DNA contamination in the RNA template, while the 5 × HiScript qRT SuperMix II was used to ensure the integrity of the cDNA by terminating the action of the gDNA wiper. The amplification of GPX3 and GAPDH was performed using qRT-PCR. In the reaction system, 0.4 μl of forward primer (10 μM) and reverse primer (10 μM) were added individually, resulting in a final concentration of 0.2 μM. Simultaneously, the 2-∆∆CT method was utilized to determine the mRNA expression levels. The sequence is provided below:

GPX3 forward primer: 5′-GAGCTTGCACCATTCGGTCT-3′, reverse primer: 5′-GGGTAGGAAGGATCTCTGAGTTC-3′; DUBR forward primer: 5′-CTCAGGATGGACCATGAGCTT-3′, reverse primer: 5′-GGGTAAGTGCGTTGTTGGT-3′; GAPDH forward primer: 5′-TCGGAGTCAACGGATTTGGT-3′, reverse primer: 5′-TTCCCGTTCTCAGCCTTGAC-3′.

The Bulge-Loop^TM^ miRNA qRT-PCR Starter Kit (RiboBio, China) and Bulge-Loop^TM^ miRNA qRT-PCR Primer (RiboBio, China) were employed for the reverse transcription and qRT-PCR analysis of miR-502-3p and U6. U6 was utilized as an internal reference for miR-502-3p. The final concentration of the forward and reverse primers in the qRT-PCR reaction system was 5μM.

### StarBase database analysis

We conducted an analysis of 32 cancer types utilizing the TCGA dataset through the utilization of starBase, a predictive tool for miRNA targets (http://starbase.sysu.edu.cn/). The identification of upstream miRNAs for GPX3 was accomplished by employing seven target gene prediction programs. To refine the results, we selected the "2 programs" option within the "Program Number" filter. The predicted miRNAs resulting from this process were subsequently considered as potential candidates for GPX3. Furthermore, the starBase tool was employed to predict the interaction between lncRNAs and the previously selected miRNAs, utilizing the "medium stringency (>= 2)" filter in the "CLIP Data" and the "2 cancer types" filter in the "Pan-Cancer" option.

### MiRNet database analysis

MiRNet, a comprehensive database (https://www.mirnet.ca/), amalgamates multiple databases to facilitate the prediction of miRNA-target interactions. In this study, miRNet was employed to forecast potential interactions between miRNAs and lncRNAs. Subsequently, the intersection of lncRNAs derived from starBase and miRNet was utilized to construct a refined set of upstream lncRNAs with the highest likelihood of interaction.

### CancerMIRNome database analysis

The CancerMIRNome database represents the most extensive compilation of tumor miRNA expression profiles available thus far. This database amalgamates miRNA expression profiles derived from over 10,000 tumor tissues or para-carcinoma tissues across 33 cancer species within TCGA. Additionally, it encompasses approximately 30,000 circulating miRNA expression profiles obtained from plasma, serum, and exosomes. In our study, we employed the CancerMIRNome database for conducting miRNA expression analysis and survival analysis.

### Kaplan-Meier plotter analysis

The Kaplan-Meier Plotter (http://kmplot.com/analysis/), a popular Web site for survival analysis, was used to assess the correlation between gene expression and patient survival in more than 30,000 samples from 21 types of tumors to discover and validate survival-related biomarkers. We investigated the predictive values of GPX3 in GC utilizing KM plotter. The desired Affymetrix ID was “201348_at” and the gene symbol was “GPX3”. According to the median GPX3 expression value, they were divided into low expression group and high expression group. Subsequently, we conducted an analysis to examine the relationship between the levels of GPX3 expression and the survival outcomes of gastric cancer patients, specifically first-progression survival (FP; n = 640), post-progression survival (PPS; n = 498), and overall survival (OS; n = 875).

### LinkedOmics database analysis

The information of 32 cancer types is available in the LinkedOmics database (http://www.linkedomics.org/login.php). In volcano plots and heat maps, co-expression of GPX3 was calculated by Pearson's correlation coefficient. For GPX3 related differentially expressed genes, Gene Ontology biological process (GO_BP) and Kyoto encyclopedia of genes and genomes (KEGG) pathway were obtained using GSEA functional modules.

### TIMER database analysis

In the TIMER database (https://cistrome.shinyapps.io/timer/), immune cells infiltration in tumor tissues is detected using RNA-Seq data. An analysis of GPX3 expression and immune cell infiltration level in GC was carried out using the Gene module. Furthermore, tumor infiltration levels in GC were compared with different somatic copy number alterations using the SCNA module.

### Immune-checkpoint analysis

The analysis covered eight immuno-checkpoint-relevant transcripts (including SIGLEC15, TIGIT, CD274, HAVCR2, PDCD1, CTLA4, LAG3 and PDCD1LG2). We used the ggplot2 R package and pheatmap R package to assess the correlation of GPX3 with these immune-checkpoints. By using ggplot2 (v3.3.3) and ggpubr (v0.4.0) R packages, TIDE was used to predict possible immune checkpoint blockade (ICB) responses 21.

### Drug susceptibility analysis of GPX3

The chemotherapeutic response was predicted with the help of the pharmacogenomics database GDSC (https://www.cancerrxgene.org/). In order to make the prediction, the R package "pRRophetic" was used. An estimation of half-maximum inhibitory concentration (IC50) was performed using ridge regression. We left the parameters at their default settings. In order to calculate the mean expression of duplicate genes, the batch effect of combat and tissue type was taken into account.

### Statistical analysis

The statistical analysis was conducted using version R 4.0.3 or the aforementioned online database. A Wilcox test was employed to compare two groups, while a Kruskal-Wallis test was utilized to assess the significance of three groups. For results to be deemed statistically significant, the p value or log rank p value had to be below 0.05.

## Results

### Analysis of the GPX3 expression in GC

**Figure 1:**
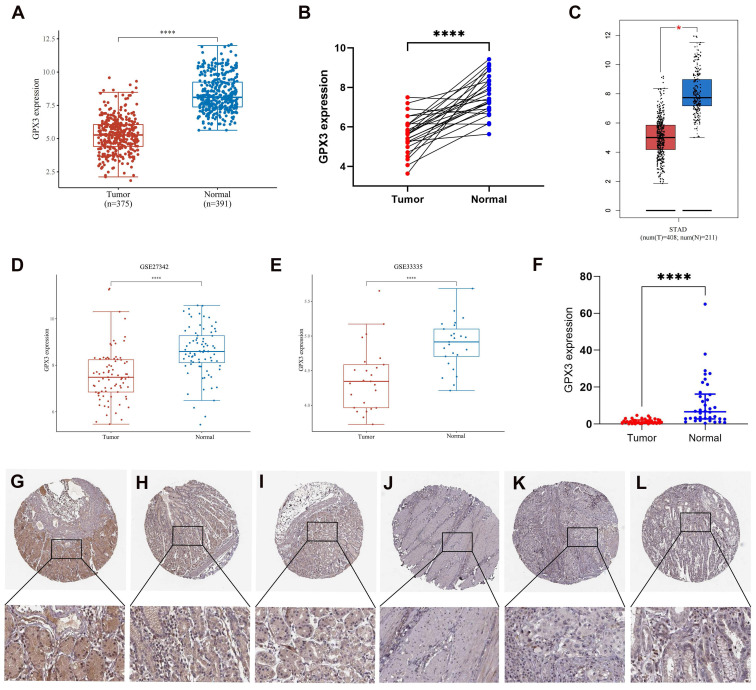
** Expression analysis for GPX3 in GC.** (A) GPX3 expression in tumor and normal tissues in GC based on TCGA and GTEx datasets. (B) GPX3 expression in 26 GC tissues and donor-matched normal tissues based from TCGA. (C) GPX3 expression in tumor and normal tissues in GC from GEPIA. (D-E) GPX3 expression in GC tissues and normal tissues from GSE27342 and GSE33335. (F) GPX3 expression in 38 pairs GC tissues and adjacent normal tissues (from Tianjin Medical University General Hospital). (D) (G-I) Representative IHC staining of GPX3 in normal gastric tissues. (J-L) Representative IHC staining of GPX3 in GC tissues. *p < 0.05, **** p < 0.0001.

In the first step, we assessed GPX3 expression in GC using TCGA and GTEx datasets. In comparison with corresponding normal samples, GPX3 was downregulated in GC (Fig. 1A). And GPX3 was lowly expressed in 26 GC tissues compared to the donor-matched normal tissues (Fig. 1B). Additional validation from GEPIA databases (Fig. 1C) and GEO databases, specifically GSE27342 and GSE33335 (Fig. 1D-E), further supported the notion that GPX3 was poorly expressed in GC. In addition, the expression level of GPX3 was quantified in 38 pairs of GC tissues and adjacent normal tissues using qRT-PCR (Fig. 1F). The findings revealed a significant decrease in GPX3 expression in GC tissues compared to adjacent normal tissues, consistent with the aforementioned data. GPX3 expression at the protein level was evaluated using IHC results obtained from HPA. As a result of the IHC staining, GPX3 expression in gastric tissues was of medium intensity (Fig. 1G-I), whereas expression in GC tissues was not detected (Fig. 1J-L).

### Prognostic value of GPX3 in GC

**Figure 2:**
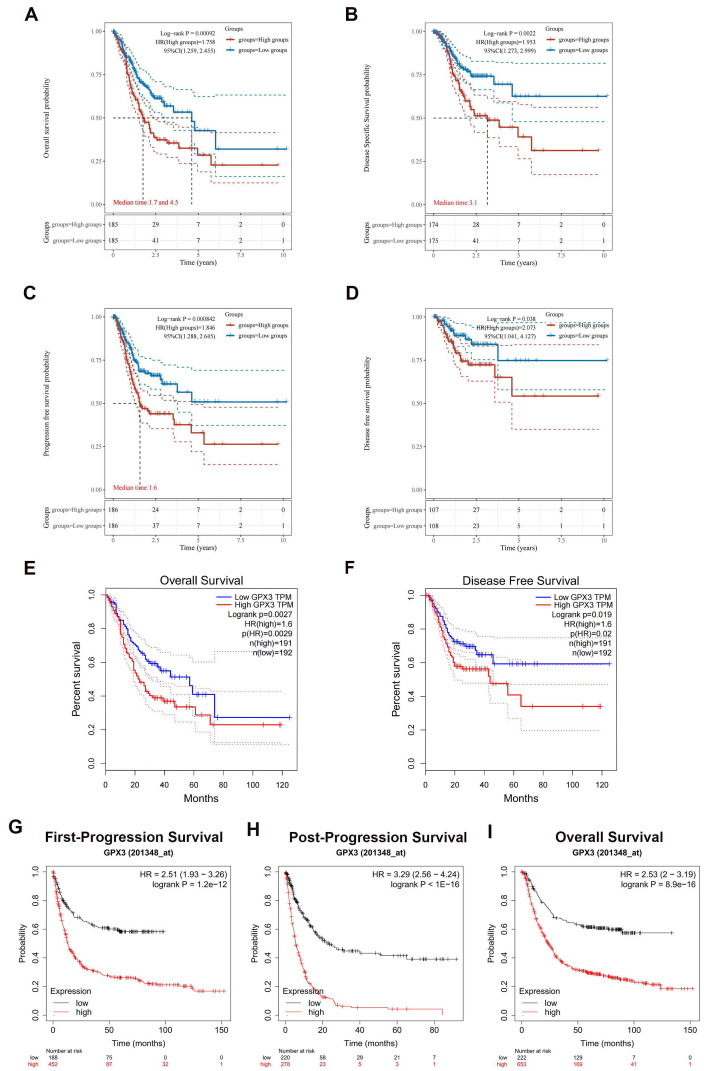
** Prognostic values of GPX3 in GC.** (A-D) A correlation between GPX3 expression and OS (A), DSS (B), PFS (C), and DFS (D) in GC based on TCGA dataset. (E-F) Analysis of OS (E) and DFS (F) of GPX3 in GC based on the GEPIA database. (G-I) Analysis of FP (G), PPS (H) and OS (I) of GPX3 in GC using the Kaplan-Meier plotter.

Subsequently, a survival analysis was conducted to investigate the relationship between GPX3 expression and OS, DSS, PFS and DFS in GC. This analysis involved the utilization of Kaplan-Meier curves to assess the correlation. The findings indicated that elevated GPX3 expression in GC patients was associated with unfavorable outcomes in terms of OS, DSS, PFS, and DFS (Fig. 2A-D). Consequently, the biomarker GPX3 holds potential as a diagnostic tool for identifying GC cases with poor prognoses. Furthermore, the association between GPX3 expression levels and patient prognosis in GC was further confirmed through the utilization of the GEPIA database and KM plotter database. In the GEPIA analysis, it was observed that increased mRNA expression of GPX3 was associated with unfavorable OS and DFS in GC (Fig. 2E-F). Then, the KM plotter tool was employed to investigate the correlation between GPX3 expression levels and patient prognosis in GC. Notably, higher levels of GPX3 expression exhibited a significant association with poor FP, PPS and OS in GC (Fig. 2G-I).

### GPX3 expression is associated with tumor progression

**Figure 3:**
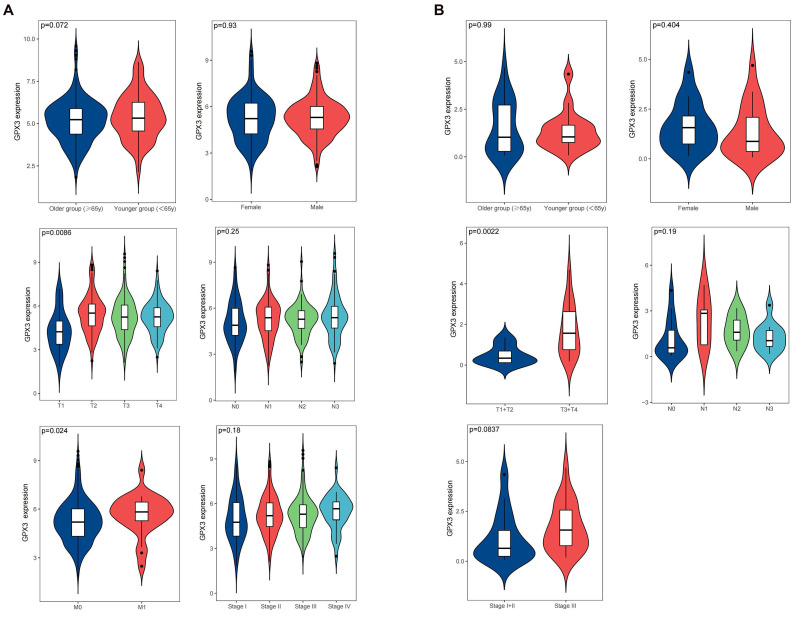
The Correlation between GPX3 expression level and clinical GC variables in the TCGA database (A) or Tianjin Medical University General Hospital (B).

The clinicopathological data of patients diagnosed with GC can be obtained from the TCGA, encompassing information such as age, gender, clinical stage, and tumor lymph node metastasis (TNM) classification. In order to conduct our analysis, we categorized the patients into two groups based on age: older group (≥ 65 years) and younger group (< 65 years). Upon analyzing this data, we discovered a significant correlation between high expression of GPX3 and T stage as well as distant metastasis, while no significant correlation was observed with age, gender, lymph node metastasis, and clinical stage (Fig. 3A). We examined the relationship between GPX3 and tumor progression in 38 pairs GC tissues and adjacent normal tissues collected. Our findings revealed a significant correlation between elevated GPX3 expression and T stage, while no significant associations were observed with age, gender, lymph node metastasis, and clinical stage (Fig. 3B). These results were consistent with the findings reported in the TCGA database.

### Prediction and analysis of upstream miRNA of GPX3

**Figure 4:**
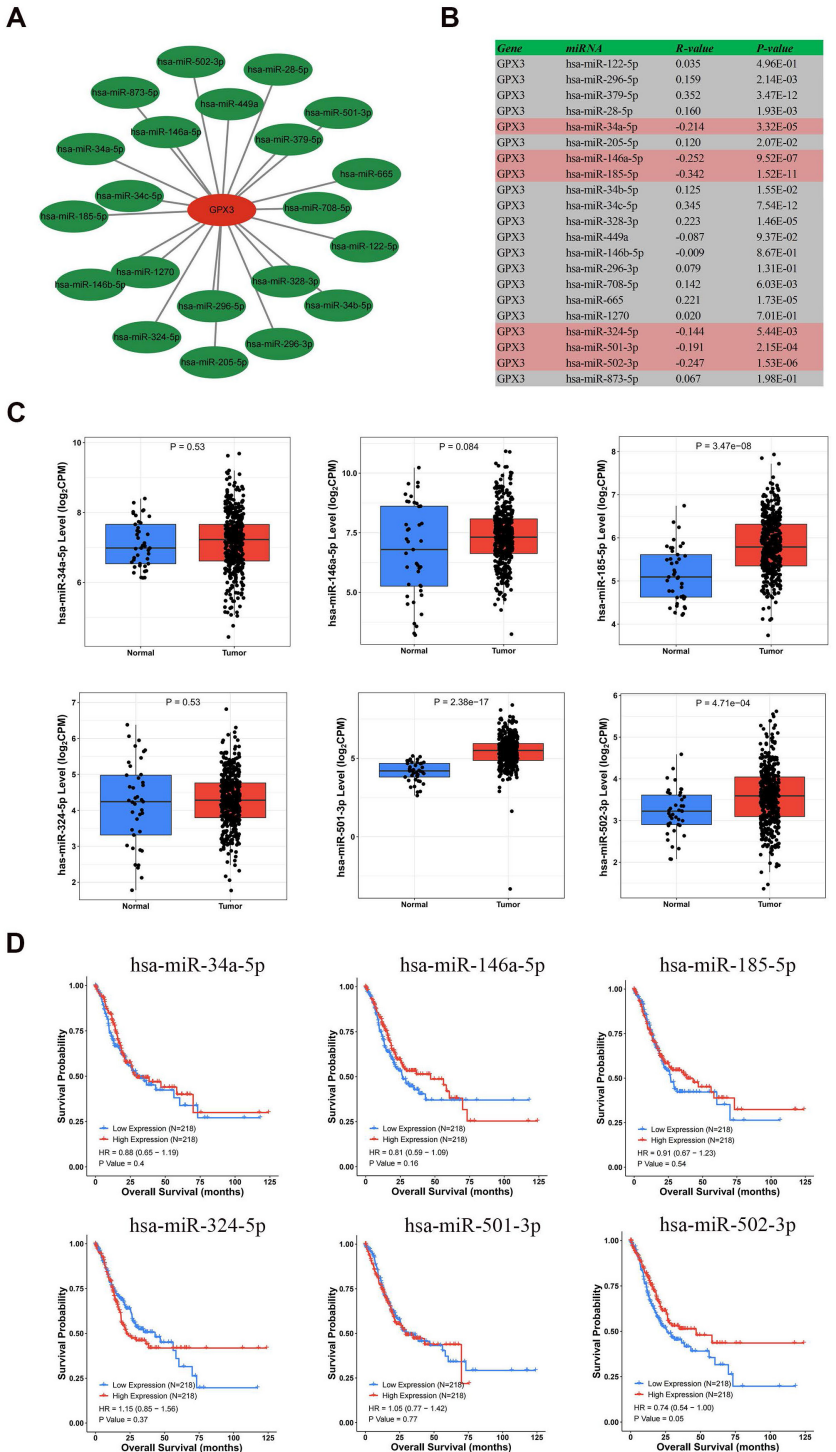
** Hsa-miR-502-3p identified as a potential GPX3 upstream miRNA in GC.** (A) GPX3-miRNA network developed by Cytoscape. (B) A correlation analysis of predicted miRNAs and GPX3 based on the TCGA dataset. (C) MiRNAs expression in GC and normal tissues based on the CancerMIRNome database. (D) A prognostic assessment of the potential upstream miRNAs in GC using CancerMIRNome database.

In order to predict the miRNAs potentially regulating GPX3, we predicted 21 miRNAs that could bind upstream of it. The data were visualized using Cytoscape software to identify miRNAs that regulated GPX3 (Fig. 4A). In accordance with miRNA's mechanism of controlling target gene expression, miRNA and GPX3 should be negatively correlated. Correlation analysis revealed significant negative correlations between GPX3 and hsa-miR-185-5p, hsa-miR-146a-5p, hsa-miR-502-3p, hsa-miR-34a-5p, hsa-miR-501-3p, and hsa-miR-324-5p (Fig. 4B). These six miRNAs were then examined using the CancerMIRNome database for their expression and prognostic value. GC patients had significantly higher expressions of hsa-miR-185-5p, hsa-miR-501-3p and hsa-miR-502-3p (Fig. 4C). Nevertheless, only hsa-miR-502-3p upregulation was associated with GC prognosis (Fig. 4D). We further examined the expression levels of hsa-miR-502-3p in 38 pairs of tissues collected from our institute, and the results revealed a significantly higher expression of hsa-miR-502-3p in GC compared to adjacent non-cancerous tissues (Supplementary Figure 1A). Additionally, an investigation was conducted to examine the correlation between the expression of hsa-miR-502-3p and TNM classification (Supplementary Figure 1B-D). The results revealed a significant increase in the expression of hsa-miR-502-3p in T1 stage compared to T3 stage. Moreover, a significant elevation in hsa-miR-502-3p expression was observed in N0 stage compared to N3 stage. These findings suggested that hsa-miR-502-3p may be associated with the T and N stage of GC. Consequently, as a possible GC regulatory pathway, hsa-miR-502-3p-GPX3 might be involved.

### Prediction and analysis of upstream lncRNAs of hsa-miR-502-3p

**Figure 5:**
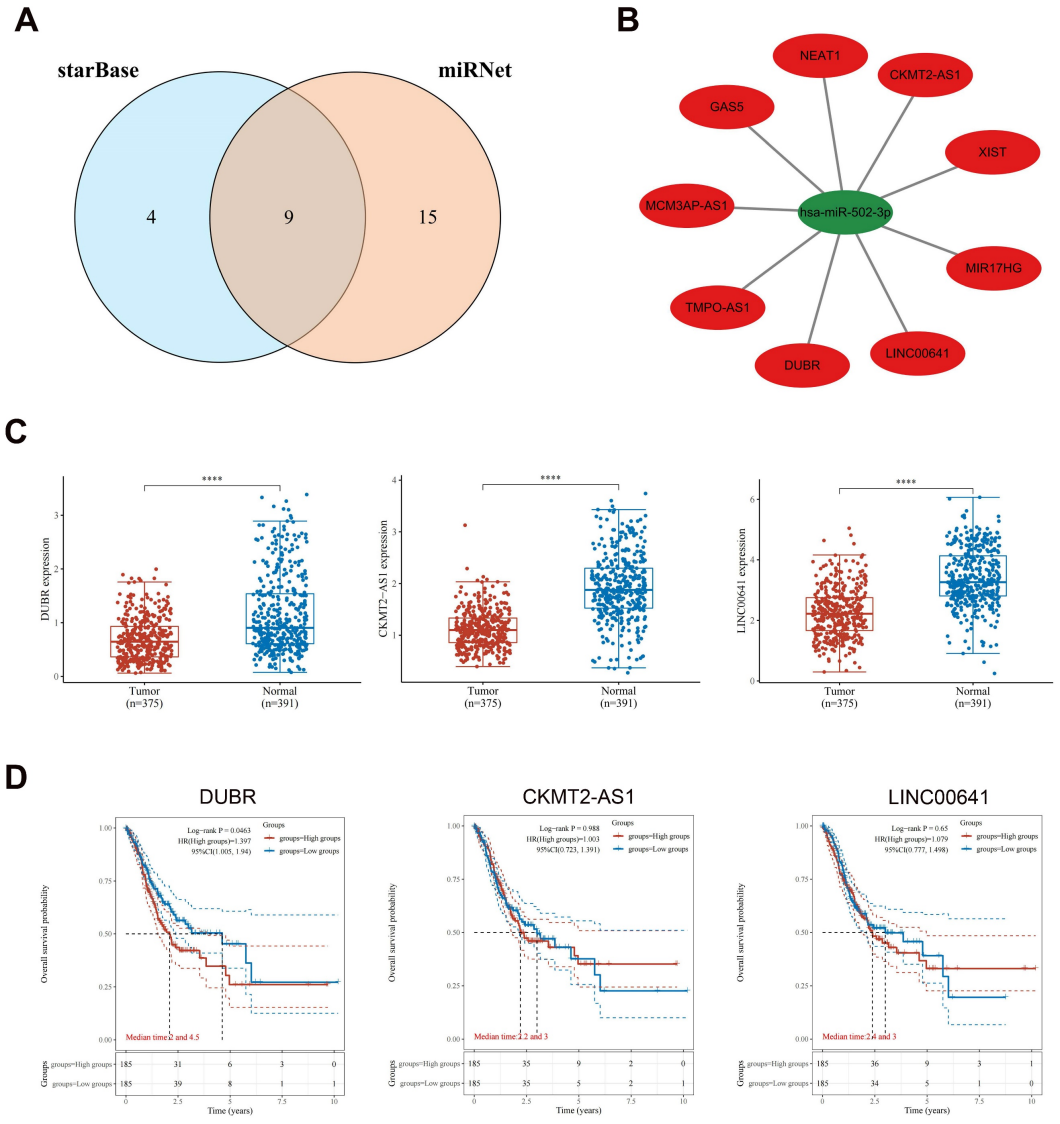
** Upstream lncRNAs of hsa-miR-502-3p analyzed for expression and survival in GC.** (A) An intersection between the starBase database and miRNet database showing potential lncRNAs associated with hsa-miR-502-3p. (B) Hsa-miR-502-3p-lncRNAs network developed by Cytoscape. (C) Potential upstream lncRNAs expression in GC and normal tissues based on TCGA and GTEx datasets. (D) The OS analysis for potential upstream lncRNAs in GC based on TCGA dataset. ****p < 0.0001.

By utilizing the starBase and miRNet databases, a total of nine potential upstream lncRNAs for hsa-miR-502-3p were identified (Fig. 5A). A similar network was constructed using Cytoscape to examine the relationship between lncRNAs and hsa-miR-502-3p (Fig. 5B). In accordance with the ceRNA hypothesis, it is expected that lncRNAs would exhibit a negative correlation with hsa-miR-502-3p and a positive correlation with GPX3. Through correlation analysis, a subset of lncRNAs that demonstrated both negative correlation with hsa-miR-502-3p and positive correlation with GPX3 was identified from the initial pool of nine lncRNAs. Ultimately, three lncRNAs, namely DUBR, CKMT2-AS1, and LINC00641, were selected as the final candidates (Table 1). As compared to controls, GC displayed significant downregulation of DUBR, CKMT2-AS1, and LINC00641 (Fig. 5C). As a result of evaluating the prognostic significance of the three lncRNAs in GC, it was determined that patients with lower DUBR expression had a better outcome (Fig. 5D). To further validate our findings, we observed a significantly higher expression of DUBR in the collected GC samples compared to adjacent non-cancerous tissues (Supplementary Figure 2A). By exploring the association between DUBR expression and TNM classification, we found a significant correlation between DUBR expression and T stage (Supplementary Figure 2B-D). As a whole, DUBR/hsa-miR-502-3p/GPX3 may be involved in GC regulation.

### The co-expression networks of GPX3 in GC

**Figure 6:**
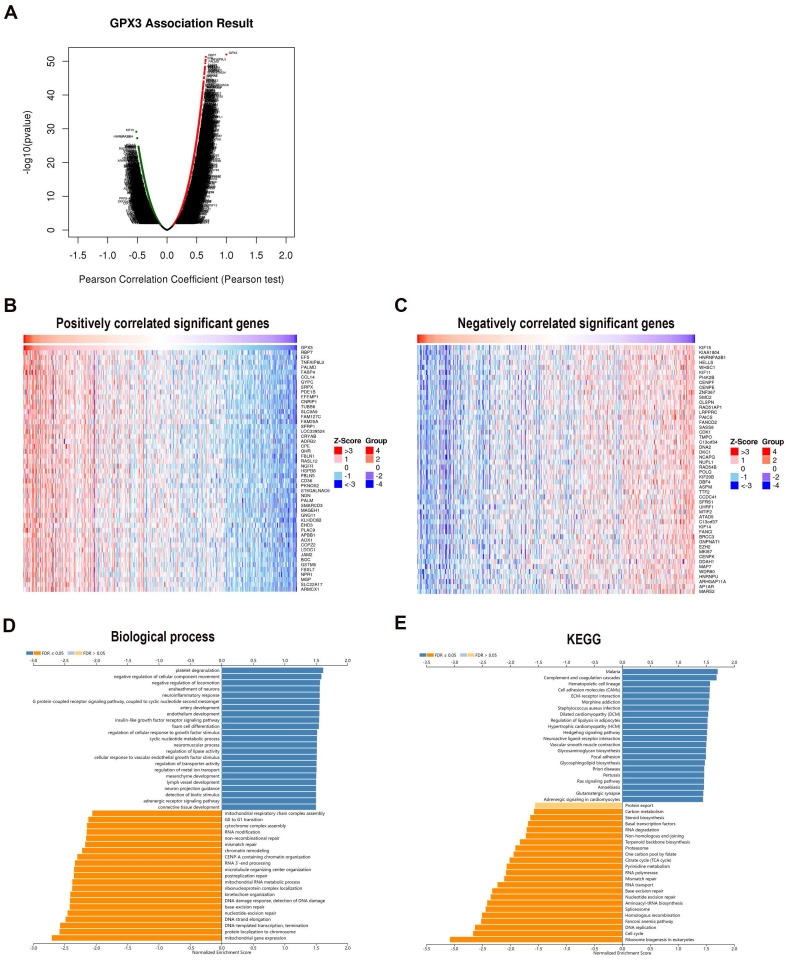
** Genes co-expressed with GPX3 in the GC.** (A) Analysis of GPX3 related genes in GC based on Pearson's test (red dots indicate positively related genes, green dots indicate negatively related genes). (B) GPX3 heatmaps showing the top 50 positive genes. (C) GPX3 heatmaps showing the top 50 negative genes. (D) GO biological processes of GPX3 co-expression genes. (E) KEGG pathway analysis of GPX3 co-expressed genes.

In order to explore the functional properties of GPX3 in GC, gene enrichment analysis was performed by using the LinkedOmics database. As indicated by the volcano map, 7512 red and 4945 green dots represented genes significantly positively and negatively related to GPX3, respectively (Fig. 6A). As well, a heat map representing the top 50 genes positively and negatively related to GPX3 in GC was provided (Fig. 6B-C). Notably, RBP7, EFS, and TNFAIP8L3 exhibited the highest positive correlation with GPX3, whereas KIF15, KIAA1804, and HNRNPA2B1 displayed the strongest negative correlation.

By analyzing GO biological process data by GSEA, SNCA co-expressed genes were connected to platelet degranulation, negative regulation of cellular component movement and negative regulation of locomotion (Fig. 6D). Meanwhile, mitochondrial gene expression, protein localization to chromosome, DNA-templated transcription, elongation and termination were inhibited. By using GSEA, KEGG pathway analysis revealed enrichment in complement and coagulation cascades as well as cell adhesion molecules and ECM-receptor interactions (Fig. 6E). These results suggested a possible effect of GPX3 on coagulation cascades and cell locomotion.

### Correlation of GPX3 with immune cell infiltration in GC

**Figure 7:**
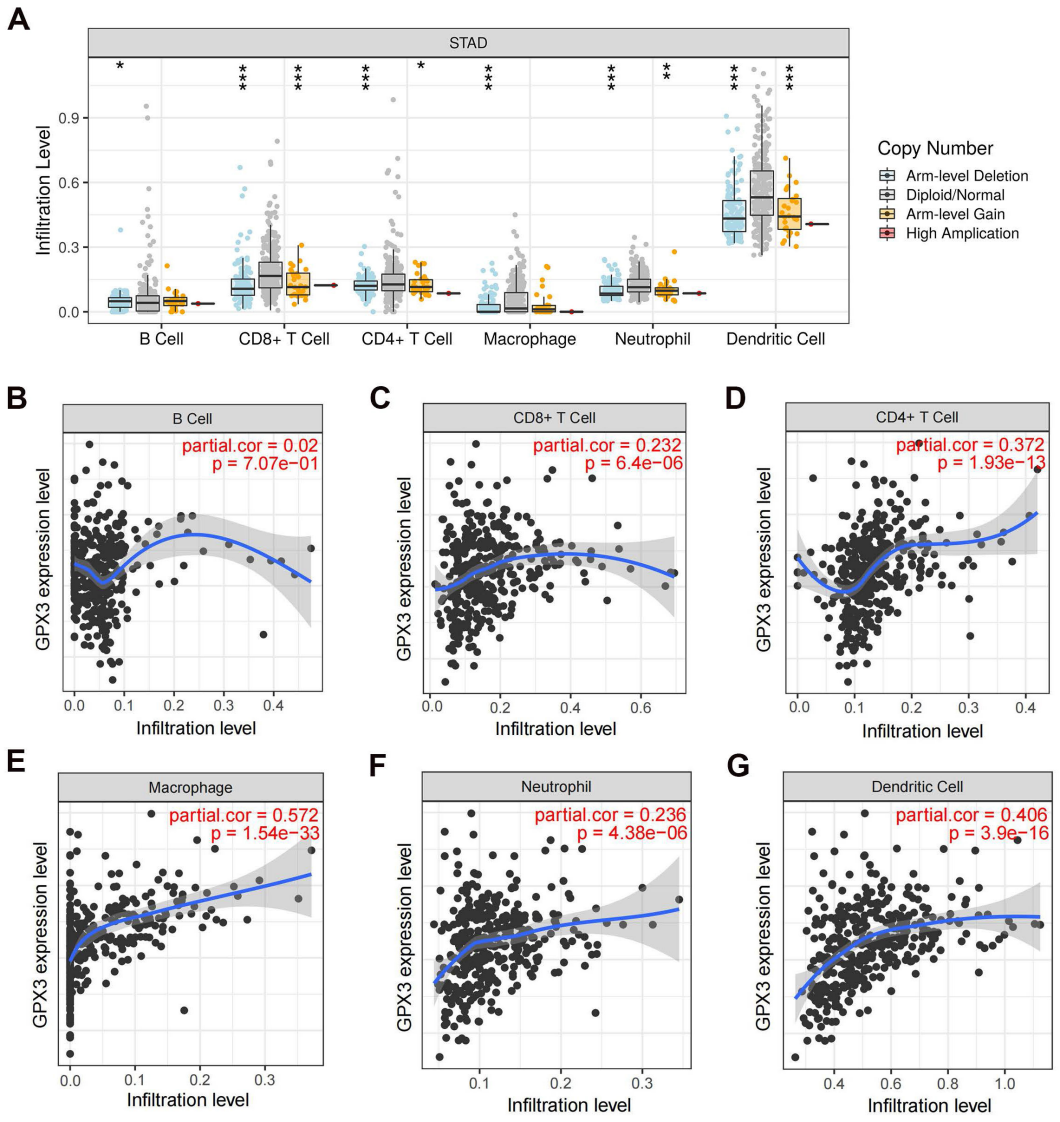
** The relationship of immune cell infiltration with GPX3 level in GC.** (A) The level of infiltration of various immune cells under different GPX3 copy numbers in GC. (B-G) The correlation of GPX3 expression level with B cell (B), CD8+ T cell (C), CD4+ T cell (D), macrophage (E), neutrophil (F), and dendritic cell (G) infiltration level in GC. *p < 0.05, **p < 0.01, ***p < 0.001.

Previous research has demonstrated a strong association between GPX3 and immune function 22,23. In this study, copy number alterations (CNAs) of the identified GPX3 features, including arm-level deletion and arm-level gain, have been found to significantly impact the infiltration levels of B cells, CD4+ T cells, CD8+ T cells, neutrophils, macrophages, and dendritic cells in GC (Fig. 7A). By examining how GPX3 expression levels correlate with immune cell infiltration levels, further research was conducted to understand its function and mechanism. A positive correlation was observed between GPX3 expression and CD8+ T cells, CD4+ T cells, macrophages, neutrophils, and dendritic cells, except for B cells (Fig. 7B-G).

### The expression correlation of GPX3 with biomarkers of immune cells and immune-checkpoints in GC

We then examined how GPX3 expression correlates with immune cell biomarkers in GC. Based on our findings, GPX3 correlated positively with B-cell biomarkers (CD19 and CD79A), CD8+T-cell biomarkers (CD8A and CD8B), CD4+ T-cell biomarkers (CD4), M1 macrophage biomarkers (IRF5 and PTGS2), M2 macrophage biomarkers (CD163, VSIG4 and MS4A4A), neutrophil biomarkers (ITGAM and CCR7) and dendritic cell biomarkers (HLA-DPB1, HLA-DQB1, HLA-DRA, HLA-DPA1, CD1C, ITGAX and NRP1), which supported the hypothesis that GPX3 and immune cell infiltration are positively correlated (Table 2).

**Figure 8:**
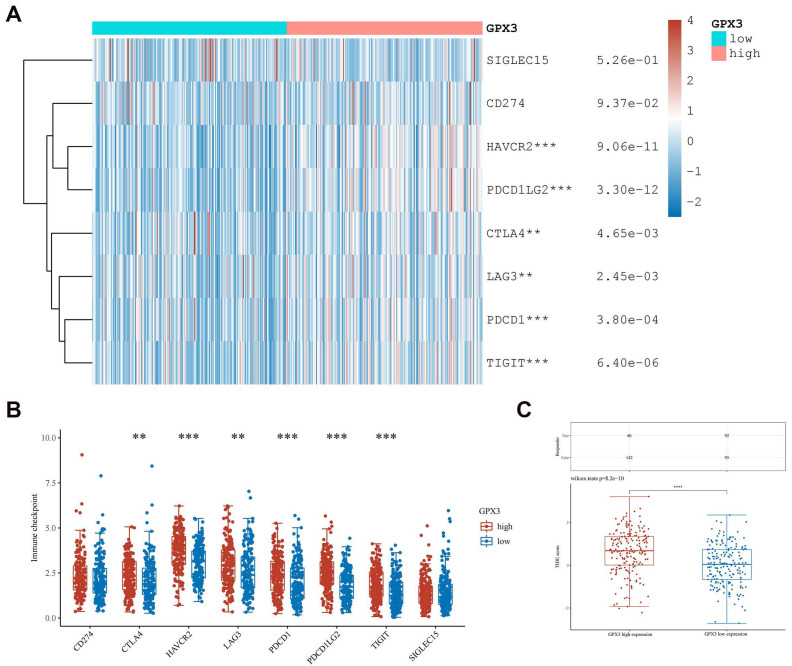
** Correlation of GPX3 expression with immune-checkpoint expression in GC.** (A-B) The immuno-checkpoint expressions in GPX3 high and low expression groups in GC. (C) The responses to ICB in GPX3 high and low expression groups in GC. **p < 0.01, ***p < 0.001, ****p < 0.0001.

The immune checkpoint in GC is also an important component of tumor immune escape, so we investigated GPX3's relationship to immune checkpoints in GC. Researchers found GPX3 expression correlated positively with HAVCR2, PDCD1LG2, CTLA4, LAG3, PDCD1 and TIGIT expression in GC (Fig. 8A-B). Furthermore, GPX3's high expression group had a higher TIDE score as compared to its low expression group. (Fig. 8C). Based on this result, the GPX3 high expression group received poor ICB therapy and had a short survival after ICB therapy. As a result of these results, GPX3 might be a potentially useful immunotherapy target for GC.

### The relationship between GPX3 and common treatments

**Figure 9:**
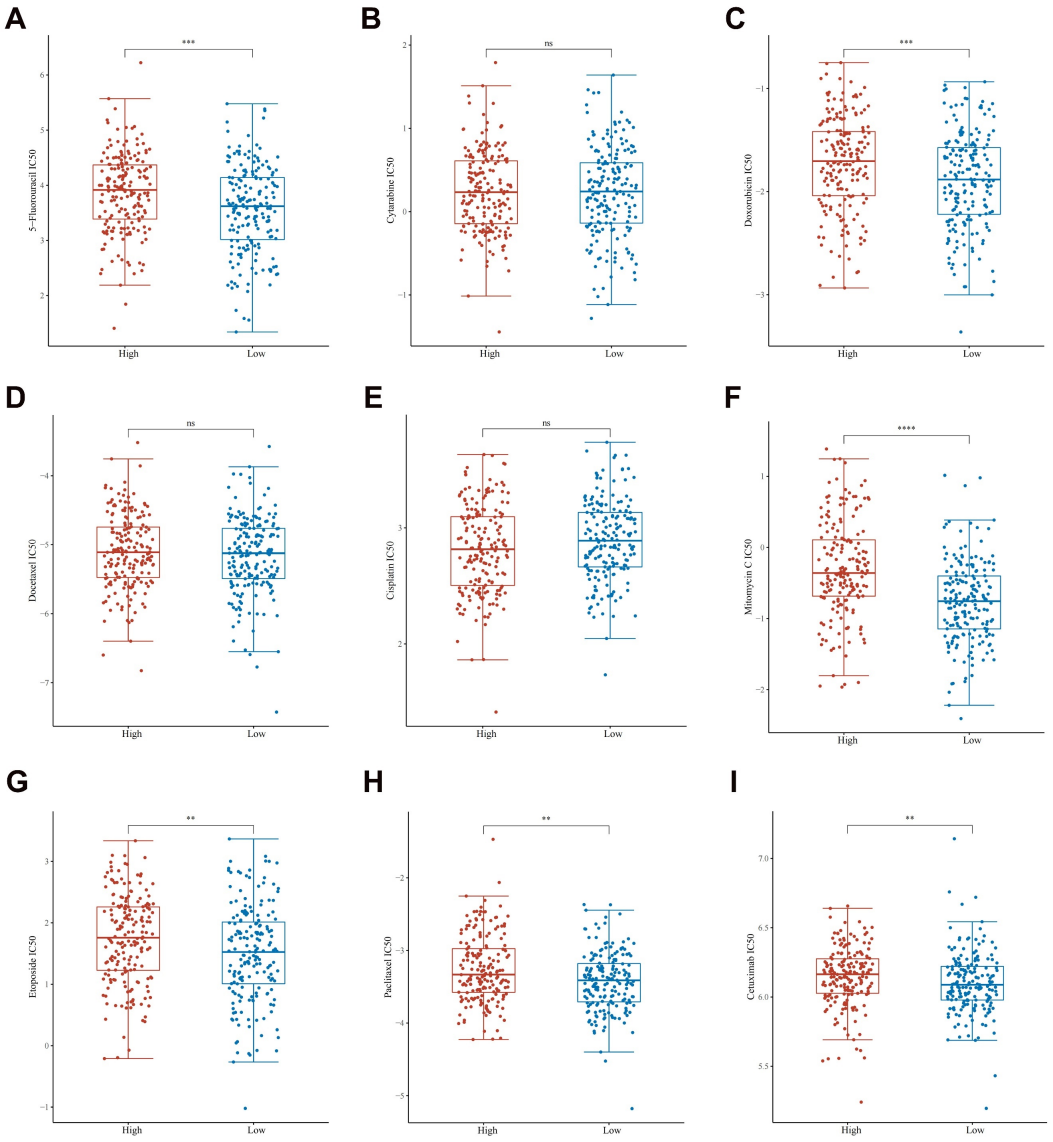
** The distribution of IC50 scores of 9 drugs in GPX3 high and low expression groups in GC.** The ordinate represents the distribution of the IC50 scores of 5-fluorouracil (A), cytarabine (B), doxorubicin (C), docetaxel (D), cisplatin (E), mitomycin (F), etoposide (G), paclitaxel (H) and cetuximab (I). **p < 0.01, ***p < 0.001, **** p < 0.0001.

In assessing a drug's efficacy or the response of a sample to therapy, the IC50 (semi-inhibitory concentration) can be used as a crucial metric. To guide the personalized treatment of GC, we compared the IC50 values of nine commonly used therapeutic agents between high expression group of GPX3 and low expression group of GPX3. We found that 5-fluorouracil, doxorubicin, mitomycin, etoposide, paclitaxel and cetuximab had significantly lower IC50 levels in GPX3 low expression groups than high expression groups (Fig. 9A-I). However, cytarabine, docetaxel, and cisplatin had no significant differences in the IC50 levels.

## Discussion

Despite the continuous improvement in drug therapy, the overall survival times for GC remain discouraging. In light of this, a better understanding of GC' s molecular markers and pathogenesis is essential. A burgeoning body of evidence indicates that GPX3 plays a crucial role in the proliferation and development of human cancers 24-30. However, the specific involvement of GPX3 in GC remains uncertain and necessitates further investigation.

In this study, the expression patterns, prognostic values, possible mechanism and relationship with immunity in GC were demonstrated. Initially, the expression levels of GPX3 in GC were assessed by utilizing the TCGA and GTEx databases, revealing a significant decrease in GPX3 expression compared to normal tissues. Subsequently, the mRNA and protein expression of GPX3 in GC were confirmed through the utilization of the GEO, GEPIA, and HPA databases. Furthermore, survival analysis of patients' prognoses provided additional evidence suggesting the potential involvement of GPX3 in GC. This study suggested that a decrease in GPX3 expression was associated with a more favorable prognosis, while an increase in GPX3 expression was significantly linked to tumor progression, deviating slightly from the prognostic patterns observed in tumor suppressor genes. Additionally, our findings revealed a significant correlation between elevated GPX3 expression and the T stage, with GPX3 expression being higher in stages T4, T3, and T2 compared to stage T1 in GC. We hypothesize that GPX3 exhibits a tumor suppressive effect during the transition from normal epithelial mucosa to early GC. However, in cells that have undergone malignant transformation, GPX3 can facilitate tumorigenesis by safeguarding them against apoptosis, which aligns with a previous study on colitis-associated carcinoma 31. This result may be explained by the fact that in GC, GPX3 increases tumor cells' capacity to cope with oxidative stress, thereby preventing tumor cell apoptosis.

The non-coding RNAs, such as miRNAs, lncRNAs, and circRNAs, are crucial in the process of tumor development through the ceRNA pathway 32. MiRNAs, which are short non-coding RNAs consisting of 19-25 nucleotides, exert their influence on protein expression post-transcriptionally by binding to the 3'untranslated region (3'UTR) of target mRNAs. Consequently, miRNAs play a significant role in the regulation of tumor cell proliferation, apoptosis, migration, and metastasis. To predict the binding of miRNAs to the GPX3 3'UTR region, we utilized the starBase website. Based on the ceRNA mechanism, miRNA has the ability to bind to the target gene GPX3, thereby impeding its translation or inducing GPX3 degradation. Out of the 19 potential miRNAs, six exhibited a negative correlation with GPX3. The CancerMIRNome database revealed that solely miR-502-3p displayed a significant increase in GC, and a higher expression level of miR-502-3p was associated with a more favorable prognosis. Consequently, miR-502-3p was considered the most probable upstream miRNA of GPX3. There has been relatively little research on miR-502-3p. Studies have demonstrated the down-regulation of miR-502-3p in gallbladder cancer, with its overexpression exhibiting inhibitory effects on the growth and metastasis of gallbladder cancer cells 33. Another study showed that in invasive pituitary adenoma, miR-502-3p was down-regulated and correlated with tumor progression 34. In addition, miR-502-3p was found to impede the proliferation and migration of GC 35. This study found that the expression of miR-502-3p increased in GC, and the higher the expression of miR-502-3p, the better the prognosis. Furthermore, we also found that the expression of miR-502-3p in T3 stage was lower than that in T1 stage. The expression level of miR-502-3p at T2 and T4 was also lower than that at T1, but the difference was not statistically significant, which may be due to insufficient sample size. Therefore, we hypothesize that the pro-tumor effect of miR-502-3p lies in its ability to promote tumor initiation and malignant transformation. However, within cells that are already malignant, miR-502-3p may gradually transform into a cancer suppressive effect, and the specific mechanism needs to be further studied.

In accordance with the ceRNA hypothesis, lnRNA binds to miRNA to inhibit its effect on mRNA 36. To further investigate this phenomenon, we utilized the starBase and miRNet databases to predict the potential lncRNAs located upstream of miR-503-3p/GPX3. Ultimately, 9 possible lncRNAs were selected. Among these candidates, DUBR emerged as the most probable upstream lncRNA based on expression levels, survival data, and correlation analysis. Notably, the lncRNA DUBR, also known as linc00883, has been previously reported to be upregulated in hepatocellular carcinoma and has been found to contribute to oxaliplatin resistance 37. Previous research has demonstrated that the expression of DUBR is down-regulated in lung adenocarcinoma and ovarian cancer, and that overexpression of DUBR hinders tumor progression 38,39. However, there is a dearth of studies investigating the relationship between DUBR and GC. In this study, it was found that GC exhibited significantly lower DUBR expression compared to normal tissues, and this lower expression was associated with a more favorable prognosis. Additionally, higher levels of DUBR mRNA were observed at the stages of T4, T3 and T2 than at the T1 stages in GC, in line with the expression and prognostic pattern of the GPX3 gene. In conclusion, DUBR/miR-502-3p/GPX3 may be a potential regulatory pathway for normal gastric epithelium mucosa transforms into GC and tumor progression, which activates at different stages of tumor development to inhibit or promote cancer. It is necessary to conduct more experiments to determine the specific mechanism.

The GPX3 protein, belonging to the glutathione peroxidase family, exhibits the ability to impede lipoxygenase and diminish the generation of lipid peroxides. Consequently, this protein deactivates ROS, thereby safeguarding against DNA damage and ensuring the stability of the genome 6,8,40. Despite its relevance in the context of GC, the precise biological function of GPX3 remains inadequately comprehended. Utilizing the LinkedOmics database, potential biological functions of GPX3 were investigated. It was discovered that GPX3 was significantly associated with gene co-expressions involved in cell locomotion and coagulation cascades. Prior research has demonstrated that GPX3 possesses the capability to recycle Nitric Oxide (NO) derived from blood vessel cells and platelets 41. Diminished GPX3 activity may induce metabolic impairment of ROS, consequently reducing NO levels. This reduction in NO levels alleviates platelet inhibition, thereby increasing the likelihood of thrombosis 41,42.

Furthermore, GPX3 alterations regulate several signaling pathways, including NF-κB, Wnt/β-catenin, and JNK signaling, which influence the proliferation and metastasis of cancer cells 24,29,31. It is thought that ROS, inducing cell proliferation, are responsible for migration, invasion and metastasis 43,44. Most cancers have high ROS levels and low ROS scavengers. GPX3 effectively diminishes ROS levels in both mitochondria and plasma, thereby mitigating their detrimental effects. As a result, GPX3 assumes a significant function in the regulation of cellular proliferation and differentiation, as well as the invasiveness and motility of various cancers such as prostate cancer 25, melanoma 26, GC 29, and other cancers 45.

In recent years, tumor immunotherapy has gained significant importance in the field. The prognosis of cancer patients can be influenced by the infiltration of immune cells within the tumor 46. In this study, we categorized the samples based on CNA patterns of the GPX3 gene to examine the variations in infiltration levels of six distinct immune cell types among these groups. The findings revealed that both arm-level deletion and arm-level gain of GPX3 gene significantly impacted the abundance of immune cell infiltration in GC. Next, we conducted a more extensive examination of the association between the expression of the GPX3 gene and the abundance of immune cell infiltration. According to the findings of our study, there existed a positive correlation between the expression levels of GPX3 and the levels of infiltration of CD4+T cells, CD8+T cells, macrophages, neutrophils, and dendritic cells in GC. Additionally, GPX3 expression demonstrated a positive correlation with these infiltrating immune cells markers. Therefore, tumor immune infiltration was probably responsible for GPX3-mediated GC. Along with tumor immunity infiltration, immune checkpoint expression may also affect immunotherapy effectiveness 47. T cell overactivation is negatively regulated by immune checkpoint, which is a transmembrane protein on the cell surface. Immune checkpoints prevent the development of autoimmune diseases in normal cells. Tumor cells have immune checkpoints that block immune cells from attacking them and weaken the immune system's ability to destroy them. GPX3 co-expressed positively with immune checkpoints including HAVCR2, PDCD1LG2, CTLA4, LAG3, PDCD1 and TIGIT according to our study. Furthermore, the low GPX3 expression group responded better to immune checkpoint blocking than the high GPX3 expression group. The results suggested that GC patients with low GPX3 expression might benefit from immune checkpoint blockade, indicating that GPX3 might have immunotherapeutic potential.

Chemotherapy has been identified as a highly effective treatment for GC. Previous studies have demonstrated that the downregulation of GPX3 expression is particularly responsive to platinum and taxane chemotherapeutic agents 9,48,49. Our analysis using GDSC further supported the notion that chemotherapy drugs exhibited enhanced efficacy in individuals with lower GPX3 expression levels. From a clinical perspective, the combination of immunotherapy and chemotherapy holds promise for improving the prognosis of patients with elevated GPX3 levels. This combined approach offers multiple advantages in the context of antitumor therapy 50,51. Immunotherapy has the potential to augment patients' sensitivity to chemotherapy through the enhancement of the anti-tumor immune response. While, chemotherapy can enhance the immunomodulatory effect by increasing the immunogenicity and susceptibility of tumor cells to immune-mediated killing. This process facilitates the cross-presentation of antigens, leading to anti-tumor effects, and eradicates immunosuppressive cells within the host. To summarize, the prediction of patients' treatment response can be achieved by screening individuals with low GPX3 expression. Nevertheless, additional investigation is warranted to ascertain the impact of altered GPX3 expression on the progression of GC and how GPX3 might be a target for therapeutic intervention.

**Figure 10:**
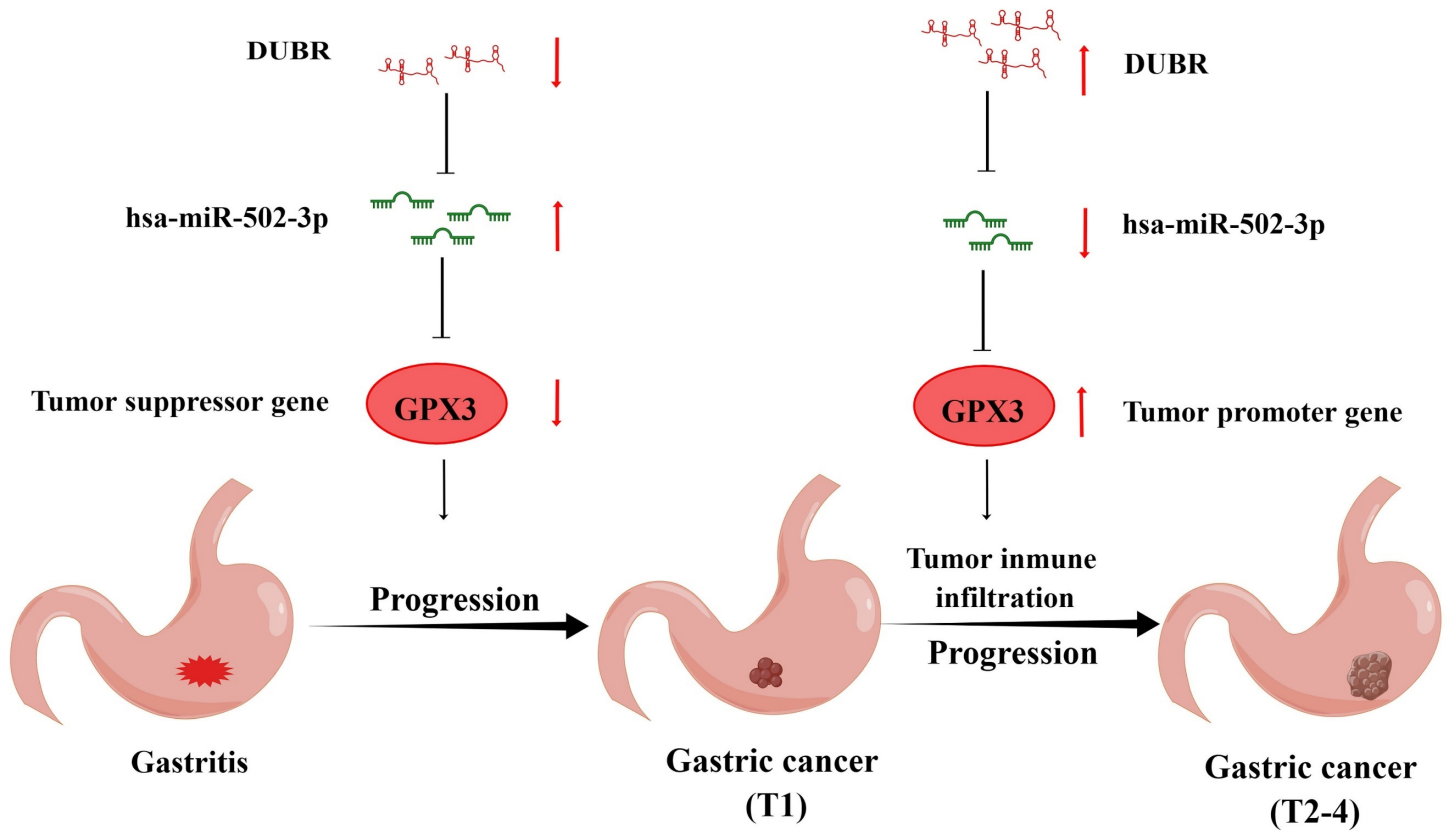
The model of DUBR/hsa-miR-502-3p/GPX3 axis of gastritis malignancy into GC and GC progression.

Taken together, we found a low expression of GPX3 in GC and an association between the higher expression of GPX3 and a poorer prognosis. For GPX3, we predicted an upstream regulatory mechanism in GC, namely DUBR/hsa-miR-502-3p/GPX3 axis, which may play a role of inhibiting in malignant transformation from gastritis to GC and play a role of promoting cancer in stepwise tumor progression (Fig. 10). Aside from that, GPX3 may contribute to cancer via increasing tumor immune cell infiltration, enhancing immune checkpoint expression, and reducing the sensitivity to chemotherapy drugs. To gain further proof, more basic studies and larger clinical trials will have to be carried out in the future.

## Supplementary Material

Supplementary figures.

## Figures and Tables

**Table 1:** LncRNAs negatively correlated with hsa-miR-502-3p and positively correlated with GPX3.

LncRNA	Gene/miRNA	R value	P value
DUBR (LINC00883)	GPX3	0.398	1.09E-15***
CKMT2-AS1	GPX3	0.250	9.82E-07***
LINC00641	GPX3	0.144	5.21E-03**
DUBR (LINC00883)	hsa-miR-502-3p	-0.401	9.07E-16***
CKMT2-AS1	hsa-miR-502-3p	-0.327	9.96E-11***
LINC00641	hsa-miR-502-3p	-0.310	1.06E-09***

**p < 0.01, ***p < 0.001.

**Table 2:** Correlation analysis of GPX3 with immune cell biomarkers in GC.

Immune cell	Biomarker	R value	p value
B cell	CD19	0.259	3.48E-07***
	CD79A	0.320	2.72E-10***
CD8 T cell+	CD8A	0.265	2.16E-07***
	CD8B	0.289	1.21E-08***
CD4 T cell+	CD4	0.429	0***
M1 macrophage	NOS2	-0.092	7.51E-02
	IRF5	0.288	1.64E-08***
	PTGS2	0.102	4.87E-02*
M2 macrophage	CD163	0.453	0***
	VSIG4	0.477	0***
	MS4A4A	0.479	0***
Neutrophil	CEACAM8	-0.051	3.25E-01
	ITGAM	0.413	6.73E-17***
	CCR7	0.412	8.61E-17***
Dendritic cell	HLA-DPB1	0.253	7.25E-07***
	HLA-DQB1	0.172	8.22E-04***
	HLA-DRA	0.197	1.27E-04***
	HLA-DPA1	0.242	2.28E-06***
	CD1C	0.455	0***
	NRP1	0.487	0***
	ITGAX	0.315	5.68E-10***

*p < 0.05, ***p < 0.001.

## Abbreviations

GC: gastric cancer
GPX3: glutathione peroxidase 3
NcRNA: non-coding RNA
ESCC: esophageal squamous cell carcinoma
lncRNA: long non-coding RNA
ceRNA: competitive endogenous RNA
mRNA: messenger RNA
ROS: reactive oxygen species
OS: overall survival
DSS: disease-specific survival
DFS: disease-free survival
HR: hazard ratios
CI: confidence intervals
IHC: Immunohistochemistry
HPA: Human Protein Atlas
qRT-PCR: Quantitative real-time PCR
FP: first-progression
PPS: post-progression survival
GO_BP: Gene Ontology biological process
KEGG: Kyoto encyclopedia of genes and genomes
ICB: immune checkpoint blockade
TNM: tumor lymph node metastasis
CNA: copy number alteration
3'UTR: 3'untranslated region
NO: Nitric Oxide
