# Supplementary material for: Prognostic value and immunological roles of GPX3 in gastric cancer
Source: Int J Med Sci. 2023 Sep 4;20(11):1399–416. doi: 10.7150/ijms.85253 (PMC10542182; doi:10.7150/ijms.85253)
Supplement: Supplementary file 1 — Supplementary figures. [file ijmsv20p1399s1.pdf]

Supplementary Figure 1. (A) miR-502-3p expression in 38 pairs GC tissues and adjacent normal tissues (from Tianjin Medical University General Hospital). (B-D) The association between the expression level of miR-502-3p and the T stage (B), N stage (C), and M stage (D) in GC using data obtained from the TCGA database. $*p < 0.05$.

Supplementary Figure 2. (A) DUBR expression in 38 pairs GC tissues and adjacent normal tissues (from Tianjin Medical University General Hospital). (B-D) The correlation between the expression level of DUBR and T stage (B), N stage (C), and M stage (D) in GC utilizing data acquired from the TCGA database. $*p < 0.05$, $**p < 0.01$.

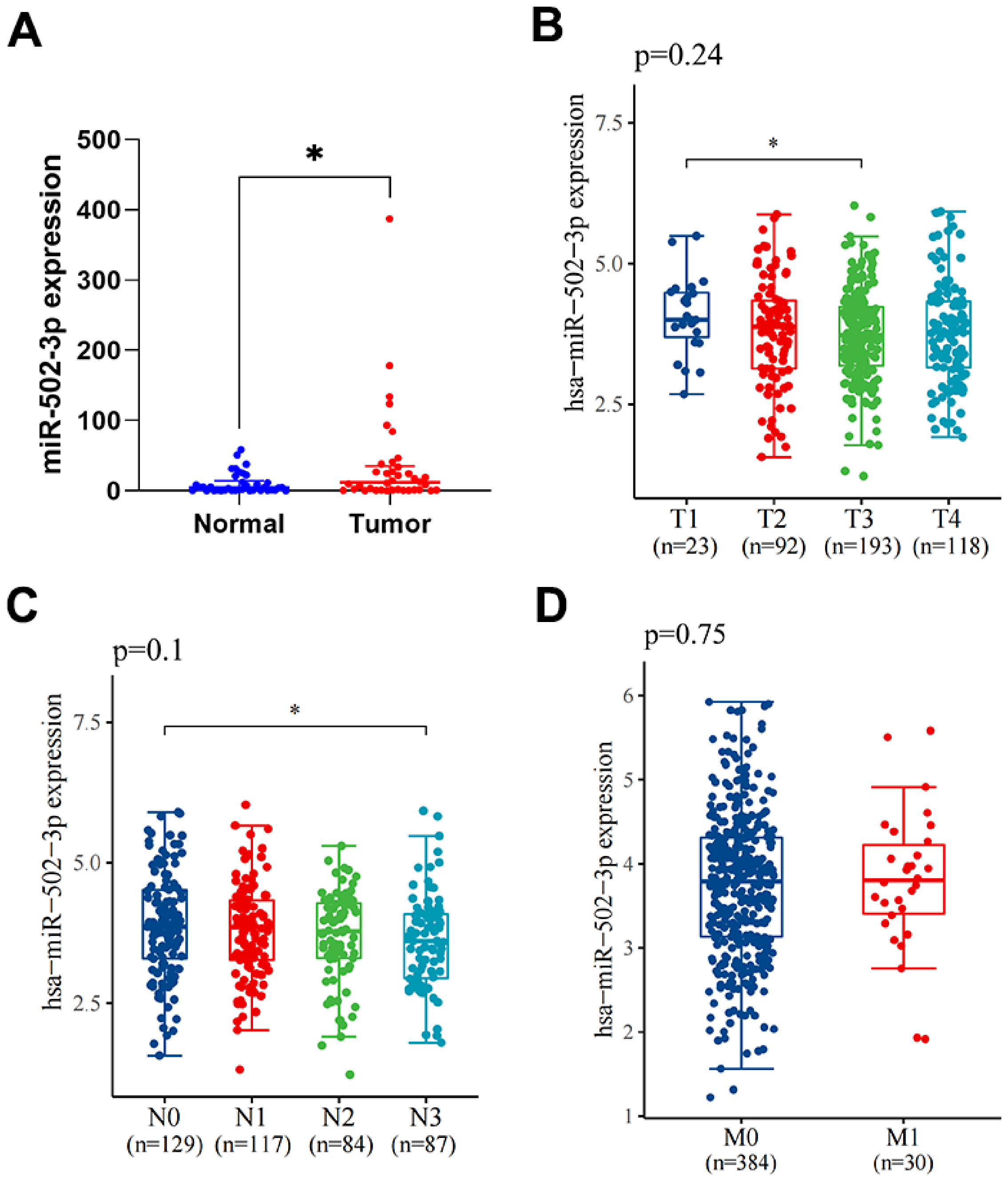

A
miR-502-3p expression
500
400
300
200
100
0
*
Normal
Tumor
B
p=0.24
hsa−miR−502−3p expression
7.5
5.0
2.5
*
T1 (n=23)
T2 (n=92)
T3 (n=193)
T4 (n=118)
C
p=0.1
hsa−miR−502−3p expression
7.5
5.0
2.5
*
N0 (n=129)
N1 (n=117)
N2 (n=84)
N3 (n=87)
D
p=0.75
hsa−miR−502−3p expression
6
5
4
3
2
1
M0 (n=384)
M1 (n=30)

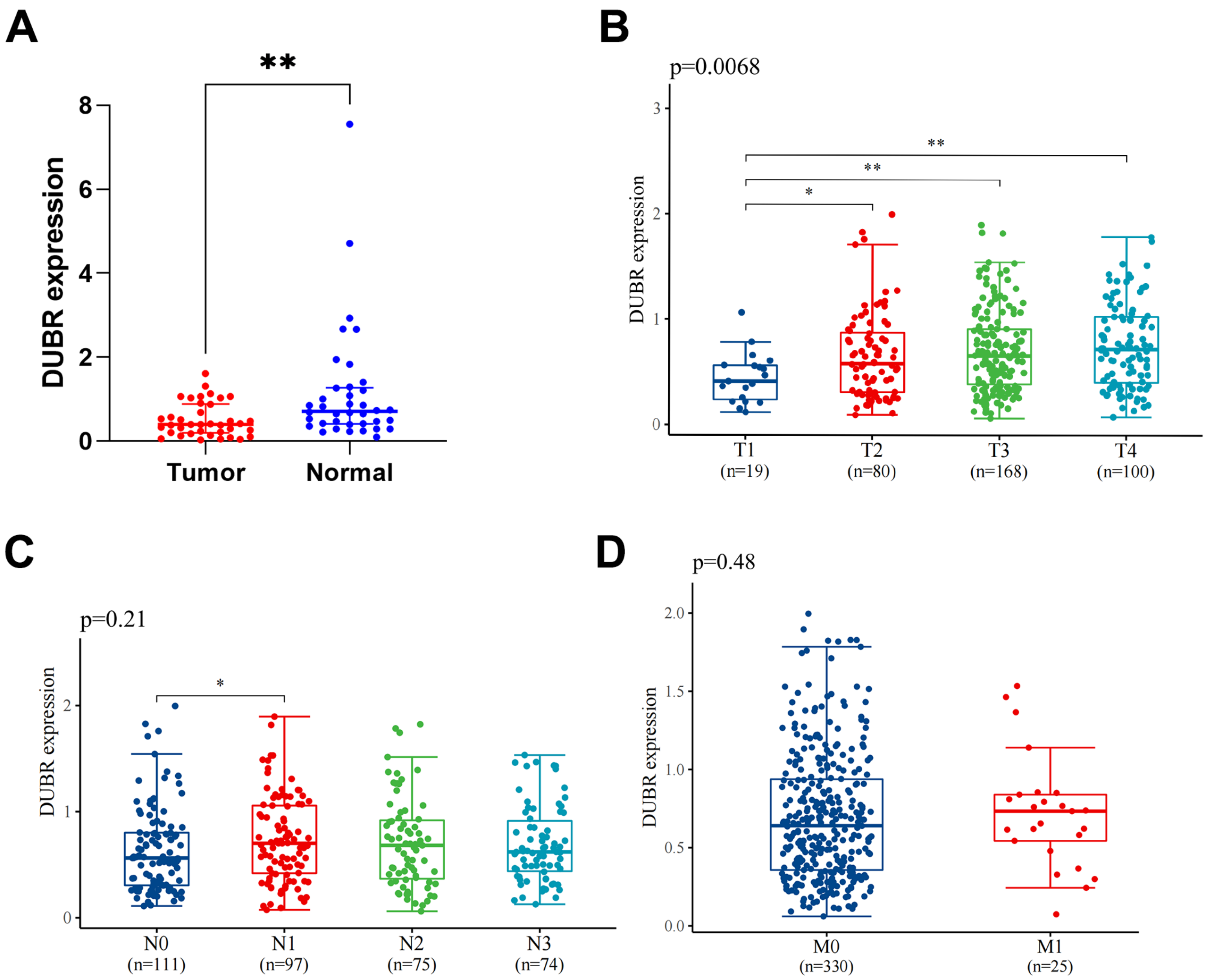

A
**
8
6
4
2
0
DUBR expression
Tumor
Normal
B
p=0.0068
3
2
1
0
DUBR expression
*
**
**
T1
(n=19)
T2
(n=80)
T3
(n=168)
T4
(n=100)
C
p=0.21
2
1
0
DUBR expression
*
N0
(n=111)
N1
(n=97)
N2
(n=75)
N3
(n=74)
D
p=0.48
2.0
1.5
1.0
0.5
0.0
DUBR expression
M0
(n=330)
M1
(n=25)
